# Ethnological Psychology

**Published:** 1856-04-01

**Authors:** 


### ART. II.—ETHNOLOGICAL PSYCHOLOGY.

ANCIENT tradition has proceeded from the East, and travelled from the rising to the setting sun. Asia was the cradle of prophecy, the nursery of wisdom, and the garden of fable, parable, and supernatural inspiration. From thence issued the most venerable writings extant, whether they be the Bible on the one hand, or Homer on the other,—the Hindoo Vedas, the Persian Zendavest, or the maxims of Confucius. Learning and fiction, divine revelation and human invention, appeared together, and flowed in a mingled, if not a turbid stream, from the Altaï and Himalaya mountains, the plains of Mesopotamia, the forests of Lebanon, the banks of the Jordan, the Lake of Gennesaret, and the coasts of Tyre and Sidon. The infancy of the world was the age of proverbs, and the spiritual apothegms of the post-diluvian epochs are now the handmaidens that wait upon the wonders of modern science. For the west and the east are two different worlds, in direct contrast to each other. Their respective voices echo and re-echo from their opposite shores, without ever blending into harmony, or even so much as becoming confused. The intelligence of the West disturbs the apathy and stolid repose of the East. The Sphynx in the sands of Egypt, and the ponderous palaces of Sennacherib at Mosul, are emblems of the mind of the people that built and beheld them. Their silence, magnitude, and monotony, smile with an air of sublimity on the fleeting generations of man and the inexorable lapse of centuries. No accordance subsists between the Asiatic and the European, no sympathy unites the energy of the one with the lethargy of the other, neither skill nor artifice can ever combine the march of intellect with the perpetual stagnation of ideas.

The notion of the three races of mankind is met with in the traditions of every people, not even excepting that of the Negroes. The first family, they say, was composed of three brothers, one of whom was black, and the other two were white. The white brothers robbed the black one of all that he possessed, and left him nothing but a little gold-dust and a few elephants' tusks. Under the names of Shem, Ham, and Japhet, the Bible rehearses the more authentic account of the three primitive stocks, and ethnology confirms the succinct narrative of the scriptures.

The Hindoos and the Persians are the twin nations that first attract our notice. Like migratory birds, fresh fledged from the tree of life, they quit their nests and fly to fairer lands that offer them a more tempting and agreeable resting-place. Thus, the Hindoo wanders along the winding beds of the Indus and Ganges, leaves the lofty mountains that hide their sources, and seeks beneath the burning sun of India those local fastnesses where he may securely indulge his love of contemplation, alone and at his ease. Listening to the rushing waters of the Ganges, Brahma ruminated in the midst of the jungles through which that river flows. But, on the contrary, the Medes and Persians flung themselves headlong down the precipitous heights of the Taurus, seized the territories where they first alighted, and made them their own. The land grew beneath their martial footsteps, the horizon enlarged in proportion to their bold advances. It was against Ahriman, the eternal enemy of their god, that they drew their swords and conquered; and, as they marched along the highways, the women quenched their thirst with a quaff from the waters of immortality. From the Persian Gulf to Armenia, and thence to the Halys, they spread themselves in battle array. Bactriana, Susa, and Persepolis, are the milestones of their journeys. Arrived at the Caucasus, they pushed onwards, until at length, under new names, but with the same spirit, they descended upon Europe. Behold the race of Japhet, as various in sentiment as in affection; armed against its own children as often as against those of others; exploring each place and thing with the strictest scrutiny, and threatening to occupy the whole globe under the well-known titles of Celts and Germans, the two-fold genius of the West!

Close by the side of the Persians and Hindoos, but almost entirely unknown to either of them, dwelt Shem in the mountainous regions around the Tigris and Euphrates. No nation ever conjoined the spirit of religion with that of industry in so remarkable a degree as the Shemites. The Chaldees, the Phenicians, the Carthaginians, and the Arabians, are of this stock, as well as the Hebrews, the peculiar people of Jehovah; and Babylon, "the Lady of Kingdoms," was the heart of the vast body of which all these several tribes were the members. The sandy desert and the ocean, the simple tent of Abraham and the ships of Tarshish, belong exclusively to this illustrious progeny, from whose sanctuary went forth in the fulness of time the gracious or appalling vocation of the Gospel.

More to the South, we perceive the race of Ham, with their black skin, curly hair, squab features, and filthy habits. They dwelt towards the centre of Africa, in those remote confines of earth where the men were said to have dogs' heads, monkeys' faces, and the ferocity of the wolf. Their spirit was as abject as their bodies. They worshipped the lion or the serpent for their god. Their social deformity shut them out from the great family of the world. Outcasts and aliens, they stretched their wigwam on the arid plains or the pestilential swamps, beneath the scorching rays of the tropics. It is supposed, that a sacerdotal mission of Hindoos brought to these wretched beings some proper notions of life and happiness; that they emigrated from Ethiopia, descended from Meroé to Thebes, and from Thebes to Memphis; that, reinforced from Arabia and Nubia, they proceeded forwards till they reached the Mediterranean Sea, and that there the superstitions, the laws, and the gods of Egypt arose and multiplied on the mud that formed the delta of the Nile.

These are the three actors that open the scene. The history of Asia is nothing more than the battle of races—Assyria, Persia, and Egypt contending for the prize. Their symbols are sculptured in relief upon the walls of Persepolis or those of Nineveh, in the forms of winged bulls with men's heads, or griffins crouching to pounce upon their prey. But the conquerors did not so much establish themselves among the vanquished, as they trod them down, till they in their turn were trodden down by the vanquished, who sooner or later rose up against them. A new feature was thus produced by these revolutions and counter-revolutions, namely, that of castes, which is the earliest sign of social inequality among men.

Another epoch of mental development occurred. Asia, teeming with her excess of population, sent forth the shepherd kings to seize upon Egypt and hold it under her sway. They modified the barbarity of the first Ethiopian colonists for awhile, but were soon expelled, and forced to seek their fortune anew elsewhere. They quitted the desert for the sea, and founded Tyre. Another emigration still more important ensued—the exodus of Israel from Egypt. Every one knows how Moses led them through stony Arabia into Palestine. The overthrow of the horse and his rider in the Red Sea was the song of triumph that still commemorates the emancipation of the soul from the thraldom of sin, and its glorious entrance into the Land of Promise. The Passover is the leading idea of the Jewish mind: it penetrates all their schemes, and peculiarizes their institutions, their habits of life, and their modes of thought. They are to this day engaged in celebrating this sublime feast, with their heads covered, their loins girded, and their staves in their hands, eating in haste, and ready to start on their mystical journey. This sentiment of transition or progression becomes a motive of action apart from the rest of mankind. They are sedate, though vagrant; a definite community, though without a settlement; merchants of wealth and credit, though destitute of a policy or emporium of their own. Of old, they were shepherds and agriculturists at one and the same time. They encamped in the wilderness; they dwelt in cities; they pitched their tabernacle in Mount Moriah, where Solomon afterwards raised the Temple, and thus rendered the worship of Jehovah no longer erratic, but fixed and concentrated in the heart of Jerusalem. In that centre were deposited the Tables of the Law, and close beside it was enacted the condign tragedy of Calvary, which sealed the fate of the Jews, and from thenceforth became the turning-point of the world. These singularities render them the most remarkable people on the face of the earth, and account for the perpetual identity of their features, their manners, and their minds.

At the same time with the exodus from Egypt took place the invasion of Greece, which was overrun by a powerful emigration from the east. It was the race of Japhet, to whom had been promised the isles of the Gentiles, as the tent and the desert had been given to Shem. The Phenicians landed in Attica, and some Egyptian adventurers crossed over to Argolis. The mysteries of Eleusis and the superstitions of Memphis lodged themselves in Parnassus. It reminds us of the Spaniards landing in Peru, or the Romans scaling the heights of Dover; only the Romans and the Spaniards were military oppressors, whereas the early Greeks were the professed friends of all they met with. They peaceably surrounded themselves with their Cyclopean walls, and marked out the site of the future city of Minerva. These marine settlers were quickly followed by others on land—a promiscuous troop that arrived at the threshold of Europe from the Taurus. But the Caucasus was the beaten path by which the main body advanced; and Prometheus is represented as being perched on the top of one of its highest peaks, and holding the east and west in either of his outstretched hands. The Danube was then, as in later times, their line of march; although, like the Goths, the greater number preferred the cheerful skies of Attica to the dreary wastes of the North. The gravest, the strongest, and the noblest of them all, were the Dorians, who debouched between Œta and Olympus, forced the isthmus of Corinth, and possessed themselves of the Peloponnesus. They drove the aborigines for shelter to the adjoining archipelago, while they strenuously closed the entrance against any further inroads on themselves.

But there was this difference between the Greeks and the Jews—viz., that the Hebrews shut themselves up within the enclosure of the Holy Land, from which they were carried off by the terrible Assyrians; and that the Greeks, after affiliating themselves with everything around them, shouted aloud, like Achilles going to battle, and aspired to the conquest of the earth. They loved the world, and the things of the world; the beautiful and the sublime were the fruits of their own genius; and they claimed glory for their own share, without a partner or a peer. Opposite as the fortunes of Shem and Japhet have been in their posterity, it is difficult to decide which of the two has produced the more lasting effects on the temporal destiny of mankind. For a time the drunken festivals of the Olympic games carried the day in a rhapsody of success, while Judah, with his hands tied behind his back, stalked as a slave in front of Nebuchadnezzar on his return to Babylon. Nevertheless, at this moment, Greece with its idols lies level with the dust; its language alone remains to attest the perfection of its intellect; and its philosophy has retired from the sight of all except a learned few. But the wisdom of captive Israel survives the wreck of time, and lives in the spirit of one who has imparted his ineffable name and title to the greater portion of the living world.

It is worthy of notice, how little Egypt either advanced or retarded the progress of affairs. With a mind cast in a particular mould of its own, it began and ended in itself. Sesostris, the Pharaohs, and the Ptolemies or Lagidæ, reflected a passing ray of light on its immutable grandeur, and the victories of Cambyses ruffled for a moment its phlegmatic calm. But nothing disturbed its mental and physical stillness. Originating in Ham, or Ammon, it ceased with Cleopatra, and was silently merged into a valuable proconsulate of the Roman Empire.

The affinities of nations may be traced in their traditions and languages, but the most striking instances are those presented by their religions. Each people alters its god to suit itself. The lusty Dorians invoked Hercules for theirs, and the Doric alliance with Etolia was the marriage of Hercules with Dejanira. If Thrace civilized Lesbos, it was to the sound of Orpheus' lyre. The colonization of Cyrene was typified by Apollo's leading a damsel in a car drawn by swans to the barren coasts of Libya. The adventures of the gods increased with the increase of popular incidents; and the Ammon, Osiris, Phtha, and Isis of Egypt became the Jupiter, Bacchus, Vulcan, and Ceres of the Greeks. The celestial staff was a small one; but its titles were numerous, and its offices unlimited. The Ionians adopted Neptune, the god of the sea, and the vagabond Pelasgi left nothing behind them but sacred blocks of unhewn stone to mark their itinerary. The Persian fire-worship was rekindled in the adoration of Apollo, the ruler of the sun; the sombre credulities of Egypt were resumed in the revels of the Dionysia; and the sensual mysteries of Phenicia were fostered anew in the more elegant and still more dissolute rites of Aphrodite. The genius of Asia revived in Greece; oriental dogmas, embellished and refined, sprung up in the west, and flourished in fashions as various as the dialects, the customs, and the districts they formed or found. The variations of Paganism were the tests of its falsity; but the uncompliant worship of Jehovah by the Jews was the stubborn demonstration of the truth of the Mosaic dispensation.

The Greek populations were complete. Let us pass over to Tuscany, whither the tide of emigration next rolled. That country was even then inhabited by the Umbrians, a Celtic people, who had descended from the north by the way of the Alps; and some Caucasians also had already arrived at the top of the Adriatic, from Illyria, and proceeded along the valley of the Eridanus or Po. The Etruscans, chisel in hand, took the same route. Half Asiatic, they sculptured the forms of birds, trees, vases, and utensils, till then unknown in Europe, and sat themselves down between the Arno, the Apennines, and the Tiber. The Sabines, the Œnotrians, and the Ochri, knew nothing of their own origin; the Dorians and Ionians never went further than the coasts; so that Italy preserved its purity of blood from the first. The East and the West met each other in the streets of Rome. The Pantheon contained the gods of every nation; and profane antiquity, which had entered within its precincts and closed its portals on itself, was transmuted into a petrifaction beneath its capacious dome.

On returning to the present state of the world, we behold three distinct races of men — the white, the tawny, and the black—as different from each other in the character of their minds as they are in the colour of their faces. Of these three, the black and the tawny are governed by the white; and of the white, the Saxons and Anglo-Normans reign supreme.

In their wild and primitive condition, the Negroes have always been an inferior order of mankind. When allowed to indulge their *natural* propensities, they are filthy and naked, painted or smeared with grease, dirty and lazy, treacherous and cruel. Some of them are cannibals, all of them heathens, and none of them trustworthy. The Papuans, tawny rather than black, are the highest in the moral scale among them, and yet the Papuans cannot but be classed with the savages. Nor is this lack of civilization owing to fortuitous circumstances, for it is their innate lot. They have always been savages in all ages; and the *wild* Negro of Africa and South America is the same now as he has always been. They hold no position whatever in universal history: the curse of Canaan has not yet been remitted—"the servant of servants thou shalt be unto thy brethren." The devoted nations of the promised land were descended from Canaan, and so were the Phœnicians and Carthaginians, who were so ruthlessly destroyed by the Greeks and Romans; and the Africans, who have been bought and sold like beasts, were also his posterity. The finger that wrote upon the wall at Belshazzar's feast points out the doom of Ham.

The blacks have, indeed, their redeeming qualities, in the possession of physical if not national virtues. Their sight, their senses of smell and hearing, their touch, their fleetness of foot, their dexterity in handling the bow and lance, their sagacity in hunting their prey, and their craftiness in catching it, are bodily endowments far more acute and perfect than are ever met with among the white or even the tawny races. They are gay and cheerful towards those who show them kindness,—gloomy and revengeful towards their real or supposed enemies; and their filial and parental instincts are both strong and exemplary. But, for all this, the negro, the NATIVE negro, is decidedly inferior to the European in body as well as in mind. The natives of Van Diemen's Land are absolutely unreclaimable; the Bosjesmans are dwarfish; the pigmies of Africa are as old as Homer. Pliny mentions their battles with the cranes for the sake of their eggs; and Strabo ironically says they built their cabins with the eggshells. At one time, 60,000 blacks were annually exported from the coast of Guinea, never more to return to their native land; and had they but had a spark of the spirit of the whites within their servile breasts, so vast a number might, in the course of two centuries, have successfully revolted, and in their turn have overrun and disputed the whole of Europe, or at least a very large and valuable proportion of the European colonies.

But Time, which in most instances is but a sorry artist, "who makes whate'er he handles worse," has done much in ameliorating the forlorn fortunes of this despised and neglected portion of the human family. Christianity, also, that subtle principle that leavens the mass of human corruption, is slowly penetrating the mind and senses of the blacks. Instances are being quoted of their improved intelligence, manifest piety, and the increasing aptitude of their talents for the finer arts, such as music, painting, and poetry, as well as for the more exact sciences, such as arithmetic and mathematics. The social virtues of order, regularity, and cleanliness are reported of those who have been trained by the labours of the various missionaries to adopt the manners and customs of civilized life. And, although many of these instances are particularized as the special gifts of individuals rather than the privileges of the tribe to which they belong, yet, upon mature reflection, we are led to conclude that their moral and intellectual welfare have changed for the better, and that the prospect of their being still more greatly improved as they continue to be more intimately mixed with the white populations is as certain as it is encouraging. Their emancipation must to some extent have operated most favourably on their instincts and habits, in the common course of events; and their proximity to or affinity with those who were once their taskmasters or tyrants, must tend to transform the wild man of the woods, the prairies, or llanos, into a human being of some pretensions to propriety and decorum. But the process is a slow one. European vices retard the noble undertaking. Ardent spirits have destroyed their tens of thousands in soul and body; and so cruel has been, on many occasions, the conduct of the whites towards the blacks, that the Negro implicitly regards the white Christian as his bitterest enemy—a murderer and a robber. These moral difficulties which are of our own creation, embarrass the hand of charity and mar the countenance of truth. The liberation and recovery of the negro-slave is one of the most interesting questions of the present day. We cannot suppose that so intelligent a people as those of the United States of America should persist in the use of slavery in opposition to the voice of the world against its practice, except from some very serious necessity, social or political, which they cannot overrule; and we await with confidence the happy moment when they shall feel themselves capable of obeying the dictates of humanity, and of proclaiming the freedom of those whom it would, if possible, have been much more prudent never to have enslaved.

The tawny races which cover more than half the globe, and are characterized by their broad shoulders, large heads, high cheek-bones, flat noses, long arms, and thin hair, constitute the Mongolian variety, that has figured so largely in the history of nations. Zenghis Khan, Tamerlane, Attila, and the Tartars, belong to this division. The conquest of China by the Moguls took place at the same time with their expeditions to the opposite quarter of the globe, which spread terror and desolation over Russia and Poland. The fierce Zenghis, the so-called lord of the nations, had been predicted, and was sent upon his dreaded mission of destruction, by the tutelar genius of his race. He traversed the world with his countless hosts. China, Thibet, Japan, the Mussulman empire of Carizmé, fell beneath his exterminating sword, which was stretched as far as the Caspian Sea. For several centuries Russia was incorporated with the government of Zipzak, Hungary was conquered, Silesia ravaged. Each of these countries still betrays its Mongolian cross-breed; but Russia, in her rapacious policy, exhibits the strongest tinge of her tawny blood. After these barbarous hordes had spared the rest of Europe, they returned upon Asia, and put an end to the Arabian Caliphate at Bagdad. The Saracens, imbued with a tawny taint, alarmed Europe from the South, and the Western powers have always watched, with the most vigilant jealousy, the restless temper of their tawny neighbours.

Their psychological character is that of unrelenting and indiscriminate bloodshed—unmitigated by any political changes or popular institutions beneficial to the human race, unmingled with any acts of generosity or kindness to the vanquished, and destitute of the slightest feelings of regard for the rights and liberties of mankind. Inflexible cruelty, selfishness, a disposition to cheat, and an absence of the tender affections, have everywhere marked their progress, and left an indelible blot upon their name in all ages. The Malays, and the greater number of the natives of the Indian Archipelago, are instances in point at this very hour. Barbarity, brutality, and even cannibalism, are their well-known qualities—the infernal instincts of their untamed nature. Their intelligence is greater than that of the blacks; but their morals are worse, and their disposition equally savage. The empires, indeed, of China and Japan prove them to be susceptible of a high degree of civilization, and even of pre-eminence in the useful and elegant arts of life; but their political and social institutions, already between 2000 and 3000 years old, remain stationary, and incapable of exercising any act of internal improvement and growth, or of external progress and aggrandizement of their own. Such as they were originated, so they remain: history informs us that Japan and China are the same now as they were at first. Their bloody commotions within, and their obtuse behaviour beyond, the limits of their empires, are proverbially unaltered and unalterable. They are obstinately opposed to the spirit and teaching of Christianity; and they are puzzled, as much as they are conquered, by the learning and science, the arts and arms of the whites.

The American Indians, however, show some qualities of much higher merit than their opprobrious colour might seem to claim for them; their industry, endurance, and fidelity are noble virtues; and the natives of Mexico and Peru appear to have been a people capable of fulfilling a higher destiny than that assigned to them in history. But it is incontestable, that neither the Peruvians nor the Red Indians equal the Europeans, under whose sway they invariably diminish or disappear. The Osmanli Turks, the mixture if not the source of whose blood is Circassian, possess far higher mental endowments than their inveterate foes the Russians; but the fatal creed of Mahomet chills their manners, congeals the noblest impulses of their souls, and is incompatible with freedom of thought and action.

The whites, with their oval faces and aquiline noses, ruddy complexions and fair hair, well-turned limbs and handsome demeanour, have hitherto governed the world. They are the descendants of those who entered Europe by the way of the Caucasus; the Circassians and the Georgians are esteemed their most beautiful specimens; and their attributes are typified in the statues of Apollo, Theseus, and Hercules. The colour of their skin discriminates them from the tawny or the black not more effectually than the pre-eminence of their moral feelings and intellectual capacity. The negroes and the Tartars may evince frankness, generosity, and hospitality, at times, in the highest degree; but in their general powers of knowledge, reflection, and understanding, they fall miserably below the whites. No European people has ever been in a condition similar to that of the present dark races, within the reach of any history or tradition. The whites may have degenerated, as in the cases of the Greeks and Romans; but they have always recovered themselves from their occasional failures or relapse, and their transcendent qualities have at no time been extinguished. Their natural prerogatives may be discerned in their least advanced states of civilization. The Germans of Tacitus and Cæsar were in no wise like the modern Hottentot or Red Indian; neither were the ancient Spaniard or Caledonian ever the same as the aboriginal African, American, or Mongolian tribes. The whites possess in the names of Scipio, Brutus, Virgil, Cicero, Horace, Livy, and many other equally great and gifted individuals, a galaxy of talent, not only unrivalled by the black or tawny races at their best estate, but also the representatives of their own lofty pretensions throughout all generations; and Theodosius or Charlemagne, Dante or Galileo, Torricelli or Raphael, Alfred the Great or Sir Isaac Newton, transmit the same intrinsic superiority of the race which they adorn, from one generation to another. To the Caucasians and their posterity alone belong nearly all the arts and sciences, or at least the most skilful application of them to the necessities of life. The treasures of literature and knowledge, civilization in its best and widest sense, politics and government, architecture and music, painting and sculpture, trade, manufactures, military tactics, diplomacy, steam navigation, the electric wire, the freedom of the press, the rights and liberties of man, and, above all, the Christian religion, are peculiarly and exclusively theirs. Europe has been their theatre of action from the first; and thence they have branched out and planted themselves all over the world. Wherever they have touched, there they have taken root. A new nation has grown up, endowed with the social and political virtues proper to its parent stock. They have never failed to live and flourish. Their ascendency is acknowledged paramount and supreme. Their prospects are unlimited, their hopes magnificent, their final object grand and praiseworthy. The world is theirs, and their own life, as well as the lives of others, are made over to their safe keeping, as a prey within their grasp.

The Greenlander, Laplander, and Samoïede, prove by their habits and features that they do not belong to the great European family. They owe their origin to the Mongols, and retain in the north the marks of their extraction, which we find so strongly expressed in the Chinese and the widely-different latitudes of the south. At the same time, the parent tribes are living in Central Asia, equally removed from both their offspring. We have alreaded alluded to the Russian mind, marked off, both historically and socially, from the rest of Europe by its strong Mongolian taint, acquired so far back as the age of Zenghis Khan.

It has been supposed that climate has modified, discoloured, or transformed, the original type of man. This theory is nowhere countenanced either by present facts or historical evidence. On the contrary, the tanned or sunburnt European is not the same as the African negro of the tropics; their natures are as distinct as their colours, with which climate has nothing to do; for blacks with blacks beget blacks, and whites from whites give birth to whites, under every climate and on every soil. The individual is modified for a time by the extremes of heat and cold, by intermarriage, social connexions, and local influences; but the race, and the germs of the race from which he sprang, remain intact, and reappear, the same as ever, as soon as the disturbing force is withdrawn or the primitive condition restored. The acorn never produces a willow, nor the lion a colt. The breed may be crossed, or the stock grafted afresh, from stronger or weaker species of the same kind, and the offset or progeny may be disfigured or apparently changed; but nature returns to her original type; the modifications are limited to the species alone or to the individual itself; the admixture of different kinds is resented with inherent pertinacity; the mule is born sterile, and without the continual intervention of an unnatural artifice the hybrid ceases to exist.

The differences of language are at first sight not less perplexing than those of colour; for if the colours of the skin be only three, the varieties of language seem all but infinite. We are living in the midst of the ruins of the primitive tongue. There is no longer a pure and grammatical language spoken or written by any nation at present. When the Teutonic, in the eighth century, superseded the Latin, it rendered the reconstruction of a perfect language utterly hopeless; for it upset every rule of grammar then in vogue. First of all, it struck out the middle verbs and dual number, so characteristic of the Greek: it then introduced the constant use of auxiliary verbs and indeclinable moods and tenses, extracted the particle from the tenses and moods, and reduced the number of cases from five to three. The verb no longer selected its own place in the sentence, governing and governed by its noun, but was left to take care of itself by immediately following its nominative and going before its objective. The pronoun, participle, and adjective no longer agreed with the noun in number, case, and gender, known by their terminations, apposition, and agreement; and the pronoun, which had hitherto been expressed by the final syllable of the verb, escaped from its entanglement, and stood alone. The noun and the pronoun became the leading words of the sentence; and the Runic or Gothic mind gave vent to its barbarity by a grammatical solecism or egotism. The indicative mood was preferred to the potential; and it is difficult to write or speak continuously in the subjunctive or optative in any of the modern languages. It erased all those delicate inflections of the future and conditional tenses, so accurate in the Latin, so multiform in the Greek; and it abolished, at a breath, the numberless expletives with which the Greek abounds to the torment of the critic, but which rendered so rich, redundant, precise, and explicit the language that employed them so correctly and fluently. The stubborn nature of the modern, particularly of the English, idiom is almost unequal to the effort of giving utterance to rhetoric or poetry, declamation or prose, in the same lofty style as that which once charmed or controlled the fierce democracies of Greece or Rome.

It would be carrying the object of this article too far, were we to follow up our analysis by showing that the original tongues are, like the original races, only three—the Indo-Germanic, the Malayan, and the Trans-gangetic. To these three belong all the languages now spoken by man. The European is the Indo-Germanic, the most comprehensive and complete of them all. It includes Noah and Abraham, the Pharaohs, the Chaldees, the Greeks, the Romans, and the Sanskrit. But we must come to a close; and our task will have been accomplished, and its end attained, if we have been able to show that the psychology of nations is as demonstrable and conclusive as the colour of their skins, the history of their progress, and the evidences of their relative excellence and ascendency in literature, arts, arms, and religion.

---

